# Iron-Only Metasurface Broadband Absorber for Solar Energy Harvesting

**DOI:** 10.3390/nano15161263

**Published:** 2025-08-16

**Authors:** Lejia Wu, Xin Chen, Dawei Zhang

**Affiliations:** 1 Engineering Research Center of Optical Instrument and Systems, Ministry of Education and Shanghai Key Lab of Modern Optical System, University of Shanghai for Science and Technology, Shanghai 200093, China; 2 State Key Laboratory of Ultra-Intense Laser Science and Technology, Shanghai Institute of Optics and Fine Mechanics, Chinese Academy of Sciences, Shanghai 201800, China

**Keywords:** metasurface, iron, visible spectrum, broadband absorber, solar energy harvesting

## Abstract

We investigated a metasurface broadband absorber composed entirely of iron and featuring a simple bilayer structure: a metallic iron substrate topped with an iron nanodisk-patterned layer. This absorber structure achieved over 90% absorption across the visible spectrum, with an average absorption of 97%. The designed metasurface structure had an aspect ratio of less than 1, which facilitated high-quality sample fabrication. In contrast to precious or rare metals typically utilized in visible broadband metasurface absorbers, this absorber offers a significant cost advantage. Furthermore, it exhibits polarization insensitivity and maintains a stable performance under oblique incidence over a wide angular range, making it suitable for practical applications. Additionally, the high melting point and favorable thermal conductivity of iron satisfy the requirements for solar harvesting and photothermal conversion devices. Therefore, this paper presents a highly efficient, low-cost, easy-to-fabricate, and operationally stable solution that is amenable to large-scale deployment in solar energy-harvesting devices.

## 1. Introduction

Solar energy is a clean, safe, renewable resource. Compared with wind, nuclear, and geothermal energy, it offers greater stability, safety, and abundance, providing a viable solution to the current energy and environmental crises. Consequently, the efficient harvesting and conversion of solar energy are crucial for the development of renewable energy. High-performance solar energy-harvesting devices have extensive and important applications in the fields of solar cells, heating systems, seawater desalination, and hydrogen production [1,2,3,4,5,6].

Metasurfaces are artificial subwavelength structures capable of precisely manipulating the phase, polarization, spin, and amplitude of optical fields [7,8,9,10,11,12,13,14]. Because of their ultrathinness, easy integration, and excellent performance, metasurfaces have been widely used in imaging [15,16,17,18], sensing [19,20,21], and perfect absorption [22,23,24,25,26,27,28,29]. Consequently, integrating metasurface structures into solar energy-harvesting devices can leverage their high integration density and high efficiency potential. Significant progress has been made in recent years in metasurface-based solar energy-harvesting devices. However, these devices still face several challenges. First, current metasurface absorbers predominantly utilize precious metals (e.g., gold, silver [30,31,32]) or rare metals (e.g., titanium, tungsten, tantalum, zirconium [33,34,35]). However, the high cost and limited availability of these materials hinder their large-scale implementation. Second, achieving broadband absorption often requires structures with high aspect ratios to enhance light–matter interactions. Unfortunately, fabricating such high-aspect-ratio structures requires expensive and complex manufacturing processes, which makes it difficult to produce low-cost and high-quality samples [36,37]. Third, broadband absorption typically relies on the introduction of multiple layers and intricate shapes to incorporate diverse absorption mechanisms. This complexity also causes significant fabrication difficulties and can often render such designs impractical [38,39,40].

To address these challenges, we propose a high-performance, low-cost broadband absorber that operates in the visible region. This absorber device features several advantages. Firstly, the absorber is constructed entirely from iron, which has a cost advantage over rare metals and noble metals. Secondly, the design features a simple bilayer structure and low aspect ratio, exhibits facile manufacturability, and is suitable for large-scale production. Thirdly, this device can achieve high-level absorption in the band where solar radiation energy is most concentrated. This ensures that it can meet the application requirements of solar energy-harvesting devices. Finally, the analysis on the polarization and angular responses indicates that the device is polarization-independent and wide-angle and sufficient for practical application scenarios. Consequently, the designed absorber device combines high performance with low cost, fabrication friendliness, and robust stability and offers a promising pathway toward cost-effective and practical solar-energy technologies.

## 2. Materials and Methods

**Figure 1:**
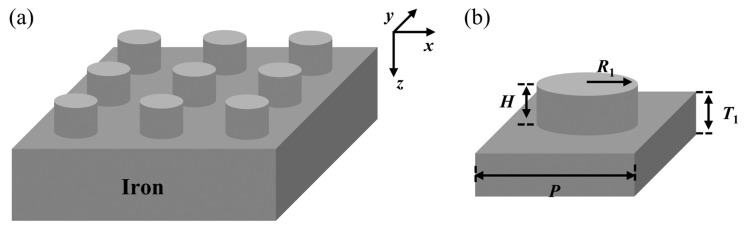
(**a**) Schematic of metasurface unit array. (**b**) Metasurface unit cell schematic and geometric parameters.

The designed metasurface structure comprised a periodic array of unit cells, as shown in Figure 1a. In each unit cell, the bottom part was a metallic iron substrate layer, and the top part was a patterned layer of iron nanodisks, as depicted in Figure 1b. The unit cell has a periodicity *P*, substrate thickness *T*_1_, nanodisk height *H*, and nanodisk radius *R*_1_. The metallic substrate was sufficiently thick to block light transmission, and the nanodisk-patterned layer further manipulated the optical field to minimize reflection, thereby maximizing structural absorption. The complex refractive-index data for iron were obtained from Palik et al. [41].

**Figure 2:**
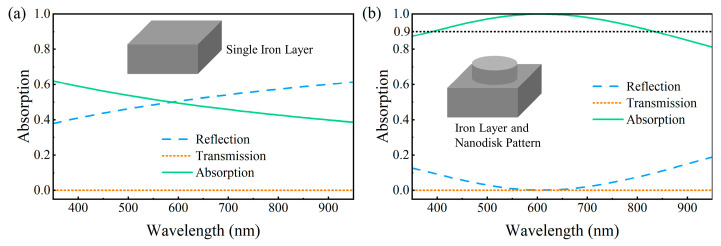
(**a**) Reflection, transmission, and absorption curves of a single iron layer. (**b**) Reflection, transmission, and absorption curves of the structure with an iron nanodisk-patterned layer added.

Finite-difference time-domain (FDTD) simulations were used to design and optimize the metasurface structures. The calculations were performed through electromagnetic simulation software Ansys Lumerical FDTD Solutions (2023 R2 version). In our simulation, the plane wave source was set to be normally incident along the *z*-direction, with the electric field direction along the *x*-axis. Periodic boundary conditions were applied along the *x*- and *y*-directions, and a perfectly matched layer boundary condition was applied along both the +*z* and −*z* directions. The transmission and reflection of the visible bands extracted from the simulations were used to calculate the corresponding absorption spectrum. Their relationships are based on Equation (1) [26,42]:

*A* (*λ*) = 1 − *R* (*λ*) − *T* (*λ*) (1)

where *A*, *R*, and *T* represent the absorption, reflection, and transmission of the metasurface absorber, respectively. As the metallic substrate completely blocks light transmission, the primary objective of our device design was to minimize the structural reflectivity. Figure 2a shows the simulated absorption, reflection, and transmission spectra of a standalone iron substrate (*T*_1_ = 200 nm). Although the transmission is nearly zero, the high-reflection characteristics result in low absorption (lower than 60%) throughout the visible spectrum. Notably, introducing an iron nanodisk-patterned layer (*P* = 100 nm, *H* = 80 nm, *R*_1_ = 40 nm) can dramatically enhance the manipulation of the optical field, as shown in Figure 2b. This structure effectively reduced the reflection in the visible region, which increased the full-band absorption to more than 90% and yielded an average absorption coefficient of 97%.

## 3. Results and Discussion

The functions and performance of metasurfaces are primarily determined by their materials and geometric parameters. Therefore, the influence of geometric features (e.g., height, width, radius, aspect ratio, duty cycle) on the performance of metasurface structures needs to be clarified. The aspect ratio (*A*_1_) and duty cycle (*D*) are defined in Equations (2) and (3), respectively.

(2) $$A_{1}=\frac{H}{2\cdot R}$$

(3) $$D=\frac{\pi \cdot R^{2}}{P^{2}}$$

**Figure 3:**
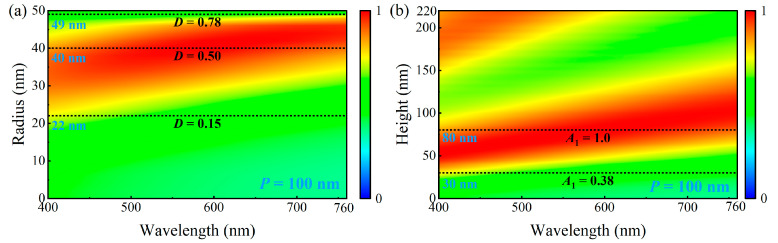
(**a**) Visible absorption spectra under different nanodisk radii of 0–50 nm with a fixed height of 80 nm. (**b**) Visible absorption spectra under different nanodisk heights of 0–220 nm with a fixed radius of 40 nm.

We systematically analyzed the absorption spectra for different nanodisk radii and heights using the parameter sweep method, aiming to investigate the influence of the iron nanodisk geometry on the absorption performance. The parameters were varied individually using a controlled variable approach, whereas the others were fixed to ensure comparable results. The nanodisk radius was varied from 0 to 50 nm, whereas the nanodisk height was limited to the range of 0–220 nm to avoid fabrication challenges owing to high aspect ratios. The substrate thickness was fixed at 200 nm. Figure 3a shows the absorption spectra for nanodisk radii ranging from 0 to 50 nm when the nanodisk height was set to 80 nm. The structure had a low duty cycle when the nanodisk radius was relatively small (the structure degenerated to a single layer when the radius was 0), resulting in weak light field manipulation and no significant improvement in the visible-band absorption. When the nanodisk radius exceeded 22 nm (corresponding to *D* > 0.15), the interaction between light and the structure was significantly enhanced, and the absorption increased accordingly. The high-absorption region (>90%) progressively extended from the short blue wavelength toward the long red wavelength, eventually covering the entire visible spectrum. However, when the radius exceeded 40 nm (corresponding to *D* > 0.5), the redshift-induced absorption reduction in the blue short-wave absorption became more apparent. When the radius approached 50 nm (corresponding to *D* ≈ 0.785), the nanodisks almost covered the entire unit cell, again degenerating into an ineffective monolayer structure and losing the absorption-enhancement effect.

Figure 3b shows the absorption spectra for nanodisk heights of 0–220 nm with a fixed radius of 40 nm. When the nanodisk height and aspect ratio were low, the structure exhibited weak optical field manipulation and no apparent absorption (degenerating to a single layer when the height was 0). When the nanodisk height exceeded 30 nm (*A*_1_ > 0.38), the interaction between light and the structure began to increase, along with absorption enhancement in the short-wavelength band; however, the absorption in the long-wavelength range remained weak. As the nanodisk height continued to increase, the absorption in the red long-wavelength band rapidly increased. When the height exceeded 80 nm (*A*_1_ > 1), the continuous red shift led to a significant reduction in short-wavelength absorption. The range capable of achieving high absorption across the entire visible spectrum was confined to a small region near the height of 80 nm.

From the above analysis, the introduction of the nanodisk-patterned layer enables more effective manipulation of the incident optical field, and the geometric parameters of the nanodisks significantly influence the absorption characteristics of the structure. Specifically, as the radius and height increased, the absorption enhancement gradually shifted from the short-wavelength region toward the long-wavelength region; however, a continuous red shift led to reduced absorption in the short-wavelength region. Balancing the absorption in the short- and long-wavelength regions is challenging. The optimal geometric parameters capable of achieving high absorption across the entire visible band were confined to a small range.

**Figure 4:**
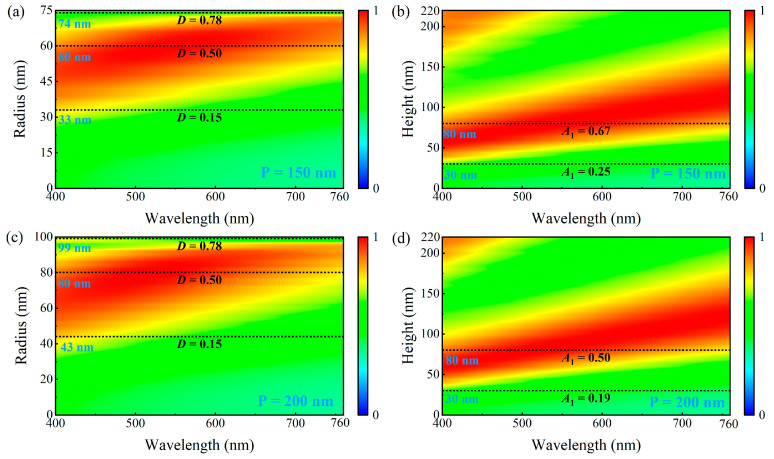
Absorption spectra as a function of nanodisk geometric parameters (**a**) with a period of 150 nm and a height of 80 nm, (**b**) with a period of 150 nm and a radius of 60 nm, (**c**) with a period of 200 nm and a height of 80 nm, and (**d**) with a period of 200 nm and a radius of 80 nm.

The unit cell period of the metasurface is also a key factor in determining the structural performance. Therefore, we studied the absorption spectra as functions of the nanodisk radius and height for periods *P* = 150 nm and *P* = 200 nm, as shown in Figure 4. Figure 4a,c show the absorption spectra for different nanodisk radii at a fixed nanodisk height of 80 nm. The different periods did not alter the fundamental absorption characteristics of the structure. With increasing radius, the absorption spectrum underwent a similar gradual shift from the short-wavelength region toward the long-wavelength region. Although the optimal radius for achieving full-band high absorption in the visible region differed for different periods (40 nm for *P* = 100 nm, 60 nm for *P* = 150 nm, and 80 nm for *P* = 200 nm), the duty cycle value for achieving the best absorption performance was the same: *D* = 0.5. The behaviors of the absorption spectra for different nanodisk heights are shown in Figure 4b,d, where the radii were fixed at 60 nm for *P* = 150 nm and 80 nm for *P* = 200 nm. The effect of height variation differed from that caused by radius changes. For different periods, increasing the radius led to a reduction in the aspect ratio at the same height (Equation (2)). However, this did not change the absorption characteristics of the structure. The optimal height value remained at 80 nm for all periods, although the aspect ratio decreased from 1 (*P* = 100 nm) to 0.67 (*P* = 150 nm) and 0.5 (*P* = 200 nm).

**Figure 5:**
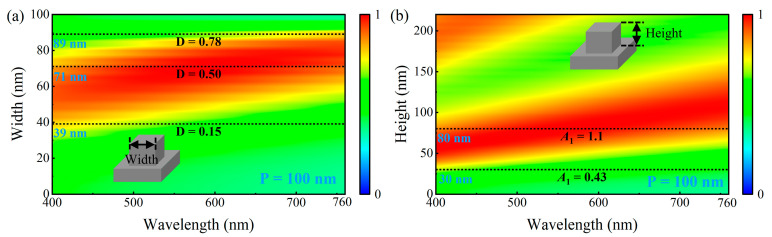
(**a**) Absorption spectra of nanoblock structure under different widths with a period of 100 nm and a height of 80 nm. (**b**) Absorption spectra of nanoblock structure under different heights with a period of 100 nm and a width of 70 nm.

Thus, we can infer that the absorption characteristics of the designed metasurface structure in the visible band primarily depend on the duty cycle and height of the nanodisk, and the optimal broadband absorption performance is achieved at *D* = 0.5 and *H* = 80 nm. The nanodisk radius and aspect ratio exhibited a weaker influence on the broadband absorption performance. To verify this inference, we studied the case in which the nanodisks were replaced with square nanoblocks with a unit cell period of 100 nm. Figure 5a shows the absorption spectra for different nanoblock widths at a fixed height of 80 nm. Compared with the nanodisk structure with a period of 100 nm in Figure 3a, the absorption spectrum of the nanoblocks shifted downward. This is because, for a nanoblock and nanodisk of equal width and diameter, the nanoblock has a larger effective duty cycle than the nanodisk. Figure 5a shows that the nanoblock structure also began to exhibit significant absorption enhancement when the duty cycle exceeded 0.15, reached the optimal absorption performance at a duty cycle of 0.5, and lost its absorption-enhancement effect when the duty cycle exceeded 0.78. This absorption behavior was consistent with that of the nanodisk structure. The absorption spectra corresponding to height variations from 0 to 220 nm with a fixed width of 70 nm are shown in Figure 5b, demonstrating that the nanoblocks achieved optimal broadband absorption performance at a height of 80 nm. This was consistent with the absorption characteristics of the nanodisks. The analysis results of the nanoblock absorption characteristics support the above conclusion. The broadband absorption of the designed metasurface structure in the visible band is highly dependent on the duty cycle and height of the pattern layer, with the optimal values of approximately 0.5 and 80 nm, respectively.

**Figure 6:**
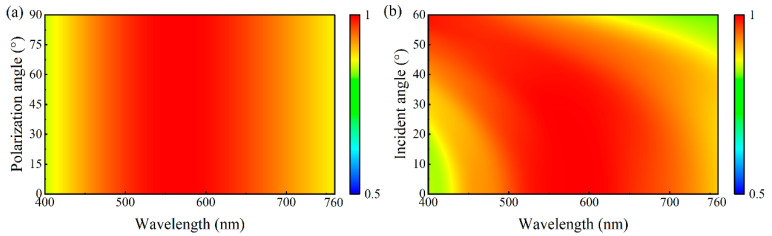
(**a**) Absorption spectra for the polarization angles of 0–90°. (**b**) Absorption spectra for the incident angles of 0–60°.

Solar energy-harvesting devices must face complex optical incidence conditions for practical applications. Natural sunlight is typically unpolarized, and the incident angle is usually uncontrollable. Therefore, polarization insensitivity and stable performance over a wide range of angles are essential prerequisites for the practical application of solar energy-harvesting devices. To evaluate these critical aspects, we examine the polarization and wide-angle characteristics of the designed absorber, as shown in Figure 6a,b, respectively. Figure 6a shows that the absorption of our structure in the visible band demonstrates a negligible change under different polarization states of the incident light, confirming its polarization-insensitive nature. Figure 6b shows the absorption spectra at the incident angles of 0–60°, demonstrating that the structure maintains a high absorption performance over this wide-angle range. Although the absorption slightly decreased in the blue waveband at incident angles < 20° and in the red waveband at incident angles > 50°, the average absorption remained above 90% across all incident angles. These results indicate that the designed absorber is insensitive to polarization and can operate effectively over a wide range of angles, thereby satisfying the requirements for practical applications.

**Figure 7:**
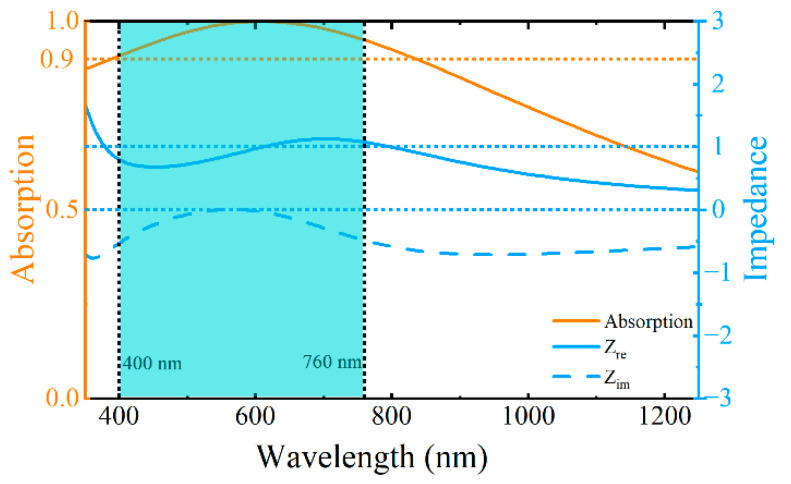
Absorption curve of the absorber from 350 nm to 1250 nm (orange solid line, axis on the left); the cyan area represents the visible band (400–760 nm). The blue solid and dotted lines on the right represent the real (*Z*_re_) and imaginary (*Z*_im_) components of the metasurface equivalent impedance, respectively.

We analyzed the absorption mechanism of the absorber using the impedance matching theory. The metasurface can be regarded as having an equivalent effect, and its equivalent impedance *Z* can be expressed as follows:

(4) $$Z=\sqrt{\frac{{\left(1+S_{11}\right)}^{2}-S_{21}^{2}}{{\left(1-S_{11}\right)}^{2}-S_{21}^{2}}},$$

where *S*_11_ and *S*_21_ are the reflection and transmission coefficients of the metasurface structure, respectively. As mentioned earlier, a sufficiently thick iron substrate completely blocks optical transmission; therefore, *S*_21_ can be ignored in the calculation. The equivalent impedance of the metasurface was calculated from the reflection coefficient, *S*_11_, as shown in Figure 7. In the visible band (highlighted by the cyan area), the real part of the equivalent impedance of the metasurface structure is close to 1, whereas the imaginary part approaches 0. This indicates that the equivalent impedance of the metasurface structure matches the free-space impedance well, enabling high-efficiency absorption. The absorption curve (orange solid line) further confirms that broadband absorption exceeds 90% across the entire visible spectrum. Conversely, in the ultraviolet (UV) and near-infrared (NIR) bands (outside the green area), the real and imaginary parts of the equivalent impedance of the structure significantly deviate from 1 and 0, respectively. This impedance mismatch between the structure and the free space leads to reduced absorption. As indicated by the orange absorption curve, the absorption markedly decreased in the UV and NIR regions. This confirms that high absorption within the visible band is achieved by the designed metasurface structure through effective impedance matching to free space in that spectral region.

**Figure 8:**
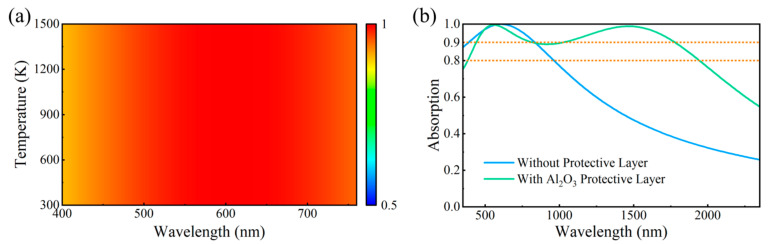
(**a**) The absorption spectrum of the absorber varying with temperature from 300 K to 1500 K. (**b**) The broadband absorption spectrum of the iron-only nanostructures without and with an Al_2_O_3_ protective layer.

In practical applications, the absorbers will inevitably be adversely affected by some environmental factors. For example, high temperatures will damage the devices, and water and oxygen will cause material denaturation and performance degradation. Therefore, high-temperature stability and oxidation resistance are important metrics for evaluating absorber performance. Thus, we simulated and evaluated the stability of the designed absorber under high temperatures. The background temperature was set from 300 K to 1500 K while keeping other parameter settings unchanged. The results are shown in Figure 8a, which shows that the absorption spectrum of the metasurface remains almost unchanged as the temperature increases, and the absorption rate remains at a high level. This indicates that the designed metasurface can stably operate in more extreme temperature environments. The well-performed thermal stability of the metasurface mainly arises from the thermal stability of iron, which is beneficial for enhancing the adaptability of the device to complex practical environments.

The designed metasurface is based on all-iron materials. Since iron is prone to oxidation in natural environments, it is essential to eliminate the adverse effects of water and oxygen on the device’s performance in practical use. Alumina (Al_2_O_3_) is a material with excellent thermal stability, chemical stability, and superior optical properties. It is widely used in semiconductors and solar photovoltaic/photothermal devices as an insulating layer or protective layer. The addition of an Al_2_O_3_ protective layer in the designed metasurface will significantly enhance the oxidation resistance and durability.

We explored the absorption spectrum of iron-only nanostructures, which are coated with a 150 nm thick Al_2_O_3_ protective layer, as shown in Figure 8b. It can be seen that the addition of the Al_2_O_3_ protective layer does not change the high absorption of the structure in the visible band and significantly extends the high absorption region to the infrared. Therefore, adding an Al_2_O_3_ protective layer to the iron-only nanostructures can eliminate the adverse effects of water and oxygen on the performance, thus ensuring that the absorber can operate stably for long-term outdoor applications.

**Figure 9:**
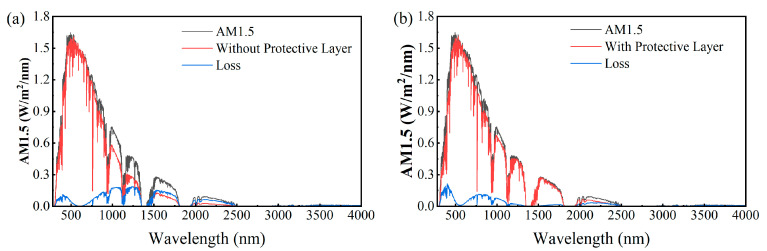
(**a**) The solar radiation energy absorption of iron-only nanostructures without an Al_2_O_3_ protective layer within the AM1.5 solar spectrum range (red curve), the AM1.5 solar spectrum (black curve), and energy loss (blue curve). (**b**) The solar radiation energy absorption of iron-only nanostructures with an Al_2_O_3_ protective layer within the AM1.5 solar spectrum range (red curve), the AM1.5 solar spectrum (black curve), and energy loss (blue curve).

Furthermore, in the practical application of solar energy-harvesting devices, it is meaningful to take the actual distribution of solar radiation energy into account. We calculated the absorption of solar radiation energy by the absorber over a broad wavelength range of 280 to 4000 nm, as shown in Figure 9. The results indicate that for the absorber without an Al_2_O_3_ protective layer, although the designed metasurface exhibits reduced absorption in the infrared band, the reduction does not significantly lower the absorber’s overall absorption across the entire solar radiation band (280–4000 nm), owing to the fact that solar radiation energy is mainly concentrated in the visible band. The average solar radiation energy absorption rate over the entire band reaches 86%. For the absorber coated with an Al_2_O_3_ protective layer, the introduction of the Al_2_O_3_ layer enhances the absorber’s absorption in the infrared, increasing the average absorption of solar radiation energy over the entire band to 92%. From the above analysis, it can be seen that the absorption of both cases is maintained at a high level within the AM1.5 solar spectrum range, which can meet the requirements of practical applications.

During the actual manufacturing of micro–nano devices, devices with excellent performance are typically limited by manufacturing tolerances. However, the structure designed in this work exhibits some more interesting phenomena. It can be seen from Figure 3 and Figure 4 that the absorption response of the structure shifts with changes in radius and height. If we only consider a single unit structure, deviations of its absorption response from the ideal state would be inevitable. Nevertheless, in the manufacturing process, the actual fabricated structure is an array composed of numerous unit structures. Fabrication errors lead to some of these units exhibiting a red shift relative to the ideal absorption spectrum, while others show a blue shift. However, the absorption performance of the entire array can remain constant—or even often improve—due to the integration of absorption spectra from different units. This involves a design strategy of integrated optimization, where a supercell composed of unit cells with nanoparticles of different sizes is used to achieve improved performance.

**Figure 10:**
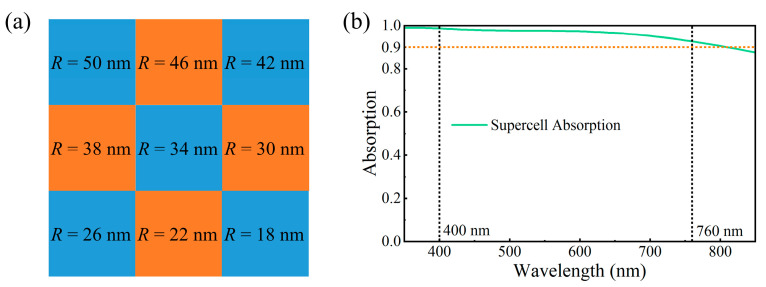
(**a**) The supercell is composed of multiple nanodisks with different radii. (**b**) The absorption spectrum of the supercell.

Furthermore, we selected nine unit cells with a period of 100 nm and different radii (*R* = 50 nm, 46 nm, 42 nm, 38 nm, 34 nm, 30 nm, 26 nm, 22 nm, and 18 nm) to form a supercell with a period of 300 nm. The arrangement of these unit cells has no particularity, as shown in Figure 10a. The absorption spectrum of the supercell is presented in Figure 10b. It can be seen from Figure 10b that the absorption rate of the supercell in the studied band not only does not decrease but also increases. The situation regarding height is similar to that of radius, as they follow a similar shift in absorption response. Meanwhile, it should be noted that the absorption response of the designed metasurface depends on the duty cycle and height of the nanodisks. It is feasible to choose a larger period (Figure 4) to provide double guarantees for the actual manufacturing tolerances. Therefore, the tolerance of the designed metasurface in actual manufacturing will be relatively high.

Benefiting from the designed absorber’s simple structure and low aspect ratio, it can be practically fabricated through various mature micro–nano manufacturing processes, such as photolithography, two-photon 3D printing technology, and electron beam lithography [17,43,44]. Among these, photolithography is a mature manufacturing technology that enables high-quality and high-throughput fabrication, facilitating large-scale applications of the absorber. Taking advantage of the simple bilayer structure and high tolerance, a standard flowchart of the photolithography fabrication, including iron deposition, photoresist coating, lithography, iron deposition, and photoresist removal, will effectively achieve the high-quality fabrication of our designed structure.

A comparison with published works is presented in Table 1. It can be seen from Table 1 that broadband absorbers often utilize rare metals and noble metals and achieve broadband absorption responses through the stacking of multilayer and intricate structures. This work adopts an all-iron design, which is cheaper and more readily available compared to rare metals and noble metals, thus offering a cost advantage. In contrast to multilayer structures, this work employs a simple two-layer structure consisting of a substrate and nanodisks, avoiding the manufacturing complexities caused by intricate structures and multilayer stacking. Moreover, the nanodisks have a low aspect ratio, enabling large-scale and high-quality fabrication through mature micro–nano manufacturing processes. Although the bandwidth of this design is narrower than that of published works, its high-level absorption covers the main region where solar radiation energy is concentrated, with a solar radiation energy absorption rate of up to 86% or even higher across the entire solar radiation band (AM1.5). This meets the requirements of solar photothermal conversion devices for high absorption, low cost, and ease of fabrication.

## 4. Conclusions

In this paper, we proposed an all-iron metasurface broadband absorption device operating in the visible spectrum. The absorber had a simple bilayer structure. Our design and performance analyses demonstrated that the introduction of iron nanodisks enhanced the interaction between light and the devices, effectively suppressed structural reflection, and achieved significant absorption enhancement. An analysis of the geometric parameters of the device revealed that the designed metasurface structure had an aspect ratio below 1, facilitating high-quality sample fabrication with controlled process complexity. Through an analysis of different periodic structures and nanoshapes, we confirmed that the absorption-enhancement effect is governed by the nanodisk duty cycle and height, with the optimal broadband absorption performance in the visible spectrum achieved at the specific parameters *D* = 0.5 and *H* = 80 nm. The results under different polarization states and wide-angle incidence show that the designed absorber is polarization-insensitive and maintains a stable performance over a wide range of angles, fulfilling the practical application requirements for solar energy-harvesting devices. Importantly, the absorber was constructed entirely from iron, which is a low-cost, readily available, and thermally robust material that enables high-performance absorption (absorption of more than 90% across the entire visible band, with average absorption of 97%) while ensuring cost-effectiveness. The designed absorber provides a new solution for solar energy harvesting and photothermal conversion applications, contributing to the performance improvement and large-scale promotion of solar energy-harvesting technology.

## Figures and Tables

**Table 1:** Comparison with related reported works.

References	Structures	Materials	Range (nm)	Absorptivity	Layers
[31]	Disk	Gold	350–800	93.4%	2
[42]	Pyramid	Titanium	200–2620	98.68%	2
[45]	Multilayer Stacks	Titanium, Chromium, Tungsten, Alumina	570–3539	97%	6
[26]	Triangular	Titanium, Alumina	200–2980	97.85%	3
[34]	Multilayer Disk	Gold, Titanium, Silica, Titanium Nitride	514–2710	>90%	6
[46]	Multilayer Cylinder	Tungsten, Silicon Carbide, Silica	200–900	95%	3
This work	Disk	Iron	400–760	97%	2

## Data Availability

Data will be made available on request.
